# Total Synthesis of Cyclosenegalin A

**DOI:** 10.1002/open.202400175

**Published:** 2024-08-20

**Authors:** Anderson Arnold Aloanis, Tati Herlina, Ari Hardianto, Rani Maharani

**Affiliations:** ^1^ Department Chemistry Universitas Negeri Manado Minahasa 95619; ^2^ Department Chemistry Universitas Padjadjaran Jatinangor 45363; ^3^ Department Chemistry Universitas Padjadjaran Jatinangor 45363; ^4^ Department Chemistry Universitas Padjadjaran Jatinangor 45363

**Keywords:** Beta-turn, Cyclopeptide, Solid-phase synthesis, Solution-phase synthesis, Total synthesis

## Abstract

A Pro‐Gly‐typed cyclopeptide, cyclosenegalin A, was firstly isolated from *Annona senegalensis* and *Annona scleroderma*. In this study, we reported synthesis the cycloheptapeptide with a combination of solid‐ and solution‐phase synthetic methods. This study also compared the effectiveness of β‐turn inducer type I and II in cyclosenegalin A to facilitate the cyclization process. The synthesis of cyclosenegalin A were prepared using two different sequences of linear peptides for cyclization. First sequence employed β‐turn type I inducer (Ser‐Ala‐Val‐Thr) as turning point and second sequence employed β‐turn type II inducer (Thr‐Pro‐Gly‐Leu). The successful cyclization was obtained using the linear sequence of NH_2_‐Ala‐Val‐Thr‐Pro‐Gly‐Leu‐Ser‐OH with β‐turn type II. The final product was obtained in 8.2 % yield with PyBOP/DIEA act as coupling agent. The synthetic cyclosenegalin A were characterized with HR‐ToFMS, ^1^H NMR, ^13^C NMR, HSQC, HMBC, TOCSY, and ROESY. The synthetic product was also evaluated for its antimicrobial activity.

## Graphical abstract

Synthesis of Cyclosenegalin A were conducted in combination of solid‐ and solution‐phase synthetic methods. The cyclization process was employed two type beta‐turn in compound and compared how both beta‐turn boosts the process.

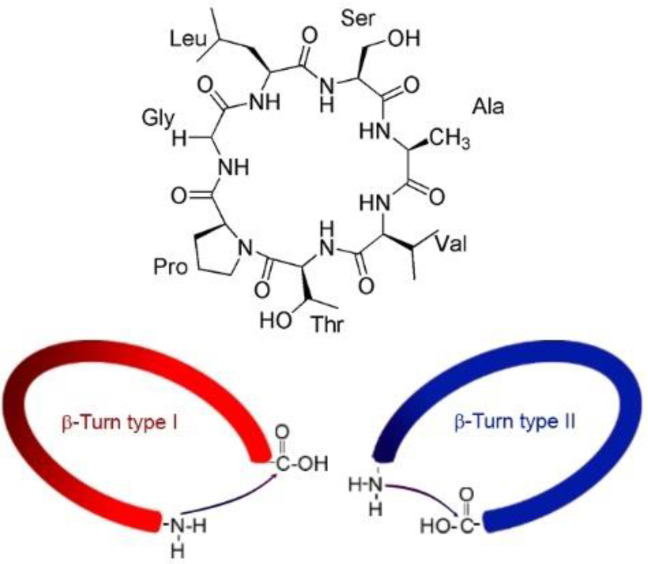

## Introduction

Cyclosenegalin A is a cyclopeptide, isolated from *Annona senegalensis* and *Annona scleroderma*.^[1]^ The peptide, cyclo(−Pro^1^−Gly^2^−Leu^3^−Ser^4^−Ala^5^−Val^6^−Thr^7^−) was isolated from the methanolic extract of both plants. Cyclosenegalin A showed activity against DU‐145 human prostate cancer cell line with IC_50_ of 54.9±2.35 μM.^[1a]^

**Figure 1:**
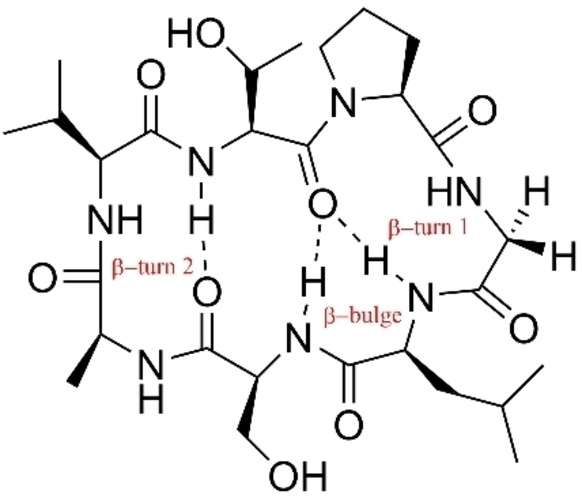
Cyclosenegalin A with two β‐turn and one β‐bulge.

Cyclosenegalin A is a proline‐glycine cyclopeptide with two β‐turns conformations, which are type I and type II, incorporating a β‐bulge (Figure 1).[^1^, ^2^] β‐turn type I involves Ser‐Ala‐Val‐Thr residues as the turning point, while β‐turn type II takes advantage of Thr‐Pro‐Gly‐Leu residues as the turning point. For these studies, each type of β‐turn was placed in the middle linear precursor to see how it facilitates the cyclization process. The synthesis was conducted using two different linear sequences. First sequence is NH_2_‐Gly‐Leu‐Ser‐Ala‐Val‐Thr‐Pro‐OH, with Ser‐Ala‐Val‐Thr as β‐turn. Ser in position *i* can stabilize the β‐turn and Ala provides stability in β‐turn.^[3]^ In addition to that, the placement of Pro residue at C terminal is a good strategy since it induces *cis* conformation and also resist to potential epimerization during carboxyl activation.^[4]^ Second sequence is NH_2_‐Ala‐Val‐Thr‐Pro‐Gly‐Leu‐Ser‐OH. This sequence places Gly and L‐Pro at *i*+1 and *i*+2 positions to form β‐turn.^[3a]^ Ser and Ala provide a combination of C‐ and N‐terminal with large and small side chains, respectively. The selected C‐ and N‐terminal was reportedly to make head to tail cyclization easier.^[5]^

In this study, we reported first total synthesis of cyclosenegalin A. The synthesis was conducted in a combination of solid‐ and liquid‐phase method. The combination method was reportedly to be effective and efficient for the synthesis of cyclic peptide.^[6]^ The linear peptide was prepared on solid phase using 2‐chlorotritylchloride resin. The solid phase strategy is known for its ability to produce high yield product with less purification step. The Fmoc/*t*Bu strategy offers mild condition during synthesis. The cyclization process was conducted in liquid phase. The head to tail amide formation was easier to achieve because the liquid phase give more flexibility than solid phase.^[7]^

In this research, our objective is to investigate the synthesis of cyclosenegalin A, which contains two types of beta turns. We aim to study the role of these two beta‐turns in the cyclization process. Additionally, due to limited information on the bioactivity of cyclosenegalin A, we also report its antimicrobial activity.

## Materials and Methods

### Material

Resin 2‐Chlorotrityl chloride (CTC), Fmoc‐L‐Pro‐OH, Fmoc‐Gly‐OH, Fmoc‐L–Leu‐OH, Fmoc‐L‐Ser(tBu)‐OH, Fmoc‐L‐Ala‐OH, Fmoc‐L–Val‐OH, Fmoc‐L‐Thr(tBu)‐OH, *N,N′*‐Diisopropylcarbodiimide (DIC), Oxyma pure, hexafluorophosphate N‐oxide (HATU), 1‐Hydroxy‐7‐azabenzotriazole (HOAt), *N,N*‐Diisopropylethylamine (DIEA), *N,N*‐dimethylformamide (DMF), dichloromethane (DCM), acetonitrile (MeCN), water (H_2_O), Piperidine, trifluoroacetic acid (TFA), triisopropylsilane (TIS), and methanol (MeOH). Amino acids, coupling reagents, solvents, and resin were purchased from GL‐Biochem, Shanghai, China.

### Method

#### Loading and capping resin.

CTC resin (0.4 g, 0.6 mmol) and DCM shaken in a SPPS reactor for 30 min to swollen the resin. The solvent was then pushed out of the reactor with pressure. The mixture of first amino acid Fmoc‐L‐Ser(tBu)‐OH (0.6 mmol) and DIEA (612.189 μL, 3,6 mmol) was dissolved in 4 mL DCM. The mixture was added to the swollen resin, followed by shaking the mixture for 4 h. After the first amino acid attached on the resin, the DCM was removed from the reactor. In order to prevent the reaction between second amino acid and resin, the unloaded active sites were treated twice using 5 mL of DIEA/MeOH/ DCM (1 : 3 : 16) for 5 min. The excess solvent was removed, and the resin was washed with DCM to obtain dry resin with the first amino acid attached. The dry resin was treated to determine the loading resin.

#### Fmoc deprotection.

The Fmoc protecting group was removed using 20 % (v/v) piperidine in 10 mL DMF for 30 min. The resin was dried and washed with DCM and DMF. The chloranil test was conducted to ensure the Fmoc protecting group was removed. Blue or red color resin indicating the existence of free amines.

#### Amino acid coupling.

Amino acid coupling was conducted with a solution of Fmoc‐amino acid/HATU/HOAt/DIEA (3 eq./6 eq./6 eq./6 eq.) in 4 mL DMF or Fmoc‐amino acid/DIC/Oxyma (3 eq./ 4 eq./ 4eq) in 5 mL DMF. The solution was added to the NH_2_‐amino acid‐resin. The reaction was shaken for 4 h. The resin was filtered and washed with DCM and DMF every time the coupling was done. The chloranil test was conducted to ensure the coupling process has completed. Unchanged resin color indicating the completed procedure.

#### Peptide cleavage.

Linear peptide cleavage cocktail was made of 2 % TFA in DCM. A 5 mL solution was added to the reactor and followed by shaking the mixture for 2 hours. The filtrate was collected and the resin was washed with 5 mL DCM three times. This step repeated two times so there will be no peptide remaining on the resin. All filtrate was combined and concentrated under vacuum to yield the crude linear peptide.

#### Peptide cyclization.

The crude linear precursor cyclosenegalin A (30 mg, 0.039 mmol) was dissolved in 39 mL DCM to produce a solution concentration of 1.0 mM. The first coupling agent used is PyBOP (3.5 eq.), with the addition of DIEA (12 eq.) and the second coupling agent used is HATU (3 eq.), with the addition of DIEA (1 %). The *t*Bu protecting group were removed with 5 mL of TFA/TIS/H2O (95 : 2.5 : 2.5). The solution was stirred in room temperature for 2 h. The solution was concentrated and washed with DCM to remove TFA residue.

#### Peptide purification and characterization.

The crude cyclosenegalin A was purified using a semi‐preparative C18 column (5 μm, 10 mm×250 mm, Jupiter) in HPLC (Waters 2998, photodiode array detector, LiChrospher 100) using combination of H_2_O and MeCN. The product was analysed using an analytical RP‐HPLC equipped with a C18 column (5.0 μm, 4.6×250 mm, Jupiter). Peptides were characterized using HR‐ToFMS, ^1^H NMR, ^13^C NMR, HSQC, HMBC, TOCSY, and ROESY.

#### Computational peptide structure prediction.

Folding prediction are performed by RPBS PEP‐FOLD4 (https://bioserv.rpbs.univ‐paris‐diderot.fr/services/PEP‐FOLD4/) developed by the Institut Pasteur Biology IT Center and the Ressource Parisienne en Bioinformatique Structurale. Accessed on 11 April 2024.^[8]^

#### Antimicrobial assay.

Cyclosenegalin A were analyzed against two gram‐negative bacteria, two gram‐positive bacteria, and one fungus. The antimicrobial assay conducted with microdilution method in a 96‐well microplate with a Mueller‐Hinton culture. The peptides were dissolved in 2 % DMSO to a concentration of 1000 ppm. Each solution was dissolved gradually. The sample solutions, positive control, and 2 % DMSO, were incubated in a 96‐well microplate at 37 °C for 18 h. The plates were read in a spectrophotometer at a wavelength of 600 nm and the MIC was calculated as the percentage of microbial inhibition.^[9]^

## Results and Discussion

The linear precursor was obtained through an initial attachment of the first residue onto 2‐chlorotrityl chloride resin. Loading resin value was determined to calculate how many first amino acid attached onto the resin. The loading resin values of first and second precursors showed 0.818 and 0.495 mmol/g, respectively. Before the second amino acid attached, the resin was capped with methanol to prevent the next amino acid reacting to the active site on the resin. After capping the resin, the Fmoc protecting group were removed from resin‐amino acid1‐Fmoc to give NH_2_ group. Then, the second amino acid were coupled to the resin‐amino acid1‐NH_2_. The first linear precursor was prepared by the employment of DIC/Oxyma coupling agent, while the second linear precursor employed HATU/HOAt as their coupling agent. The deprotection and coupling step were conducted repetitively until all the amino acids constructed on the resin. The completion of coupling and Fmoc deprotection in each step were qualitatively analyzed with chloranil test. After all amino acids coupled sequentially, the linear peptide was detached from the resin using peptide cleavage cocktail. The filtrate was collected and dry under vacuum to obtain the crude heptapeptide. The successful linear peptide synthesis in solid phase method were confirmed with HR‐ToFMS. The first precursor showed molecular ion peak at *m/z* [M+H^+^] 756.4873 (calculated C_36_H_66_N_7_O_10_ 756.4871) and the second sequence mass spectrum revealed *m/z* [M+H^+^] 756.4875 (calculated C_36_H_66_N_7_O_10_ 756.4871) (S.1–S.4). The purity of linear precursors 1 and 2 was also analyzed with analytical HPLC and both peptides were revealed at retention time 13.3 min (S.5 and S.6). The solid‐phase synthesis of precursor 1 and 2 give yields of 92 % and 96 %, respectively.

HATU and PyBOP were employed for cyclization as both reagents are widely used.[^9^, ^10^] A 30 mg of crude linear precursor was subjected to cyclization in dichloromethane. The successful cyclization was conducted for 7×24 h until all linear peptide disappeared. After the process, the solution was dried under vacuum to give crude cyclic product that was then reacted with TFA to remove the sidechain protecting group of Ser and Thr.

**Scheme 1:**
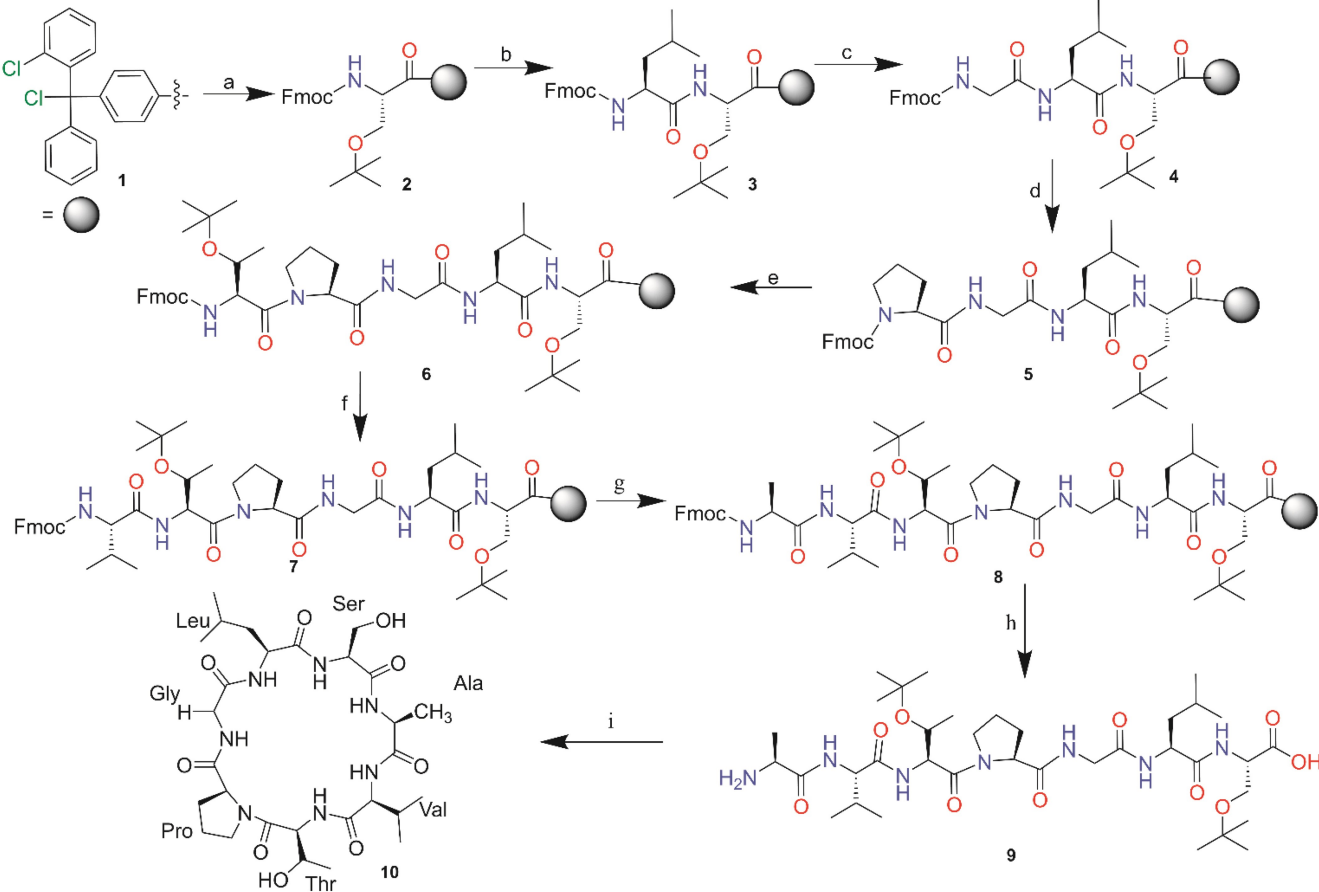
Synthesis of cyclosenegalin A from precursor 2. a). (1) Fmoc‐Ser(tBu)‐OH (1 eq), DIEA (2 eq), 4 mL DCM, 4 h, rt; (2) MeOH (15 eq.), DIEA (5 eq.), DCM (80 eq.), 30 min; (3) 20 % piperidine in DMF, 30 min. b). (1) Fmoc‐L–Leu‐OH, (3 eq.), HATU (3 eq.), HOAt (3 eq.), DIEA (6 eq.), 4 mL DMF, 4 h, rt; (2) 20 % piperidine in DMF, 30 min. c). (1) Fmoc‐Gly‐OH (3 eq.), HATU (3 eq.), HOAt (3 eq.), DIEA (6 eq.), 4 mL DMF, 4 h, rt; (2) 20 % piperidine in DMF, 30 min. d). (1) Fmoc‐L‐Pro‐OH (3 eq.), HATU (3 eq.), HOAt (3 eq.), DIEA (6 eq.), 4 mL DMF, 4 h, rt; (2) 20 % piperidine in DMF, 30 min. e). (1) Fmoc‐L‐Thr(tBu)‐OH (3 eq.), HATU (3 eq.), HOAt (3 eq.), DIEA (6 eq.), 4 mL DMF, 4 h, rt; (2) 20 % piperidine in DMF, 30 min. f). (1) Fmoc‐L–Val‐OH (3 eq.), HATU (3 eq.), HOAt (3 eq.), DIEA (6 eq.), 4 mL DMF, 4 h, rt; (2) 20 % piperidine in DMF, 30 min. g). (1) Fmoc‐L‐Ala‐OH (3 eq.), HATU (3 eq.), HOAt (3 eq.), DIEA (6 eq.), 4 mL DMF, 4 h, rt; (2) 20 % piperidine in DMF, 30 min. h). 2 % TFA in DCM, 2 h, rt. i). (1) PyBOP (3.5 eq.), DIEA (12 eq.), 7x24 h, rt; (2) 95 % TFA, 2.5 % TIS, 2.5 % H2O, 2 h, rt.

**Scheme 2:**
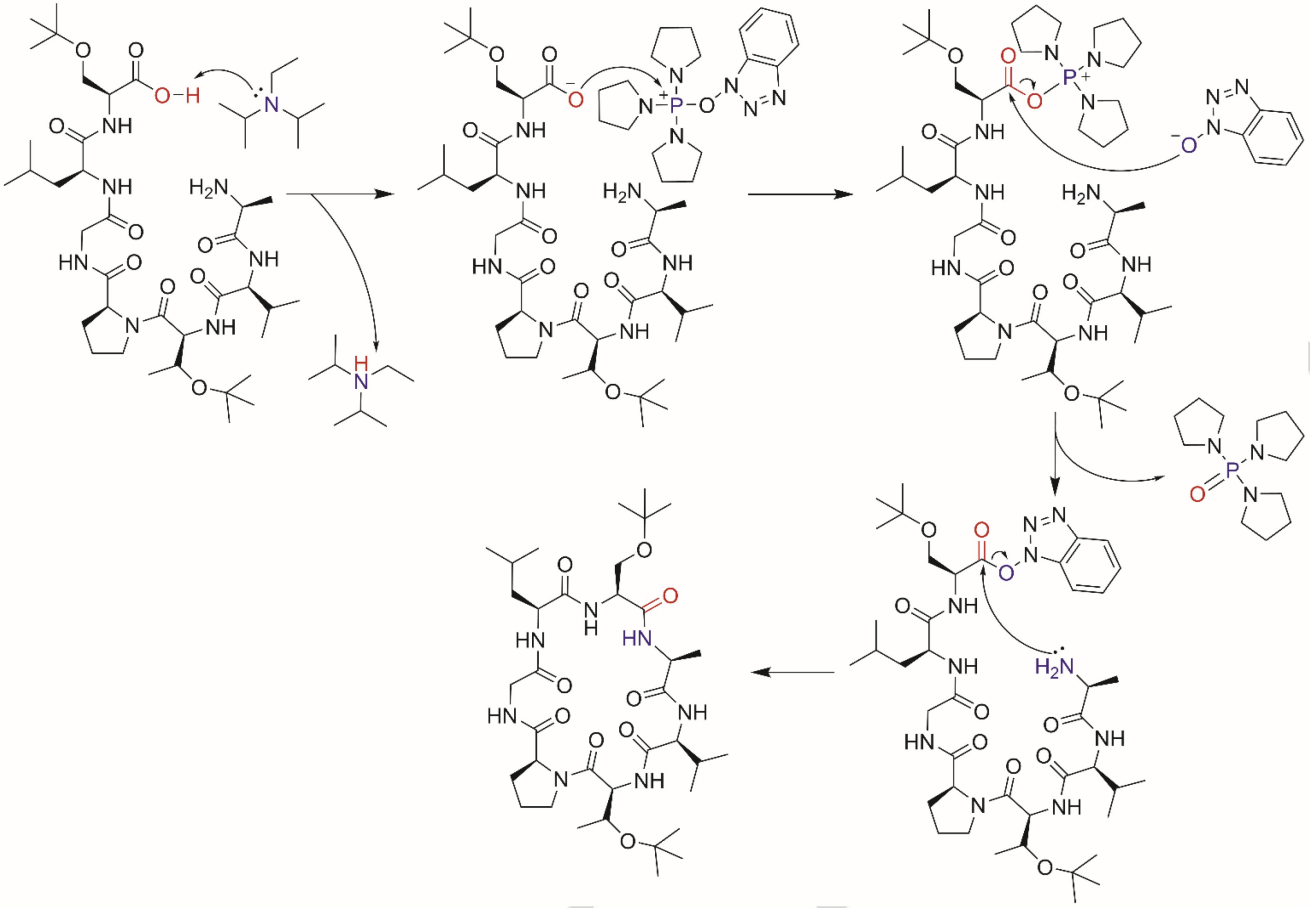
Cyclization process of cyclosenegalin A with PyBOP/DIEA as coupling agent.

A successful cyclization was observed with linear precursor 2 by employing PyBOP as the cyclization agent (Scheme 1). The β‐turn type II with Pro in position *i*+1 and Gly in position *i*+2 boosted cyclization process.^[11]^ PyBOP reagent is also known to suppress dimerization.^[12]^ β‐turn are dependent on the amino acid composition and chirality of the sequence. The unsuccessful cyclization of first linear precursor was caused by Ser and Thr in position *i* and *i*+3 that still have tBu protecting group at the side chains, and thus make the structure bulkier. The two amino acids with bulky sidechain in this position reduce the flexibility and become steric hindrance to form β‐turn.[^3^, ^11^] The cyclization reagent and condition are shown in Table 1 and the mechanism is shown in Scheme 2.

**Table 1:** Cyclization of cyclosenegalin A.

Sequence		Coupling agent	Result
1	NH_2_‐Gly‐Leu‐Ser‐Ala‐Val‐Thr‐Pro‐OH	HATU/DIEA	n/a
		PyBOP/DIEA	n/a
2	NH_2_‐Ala‐Val‐Thr‐Pro‐Gly‐Leu‐Ser‐OH	HATU/DIEA	trace
		PyBOP/DIEA	8.2 %

**Figure 2:**
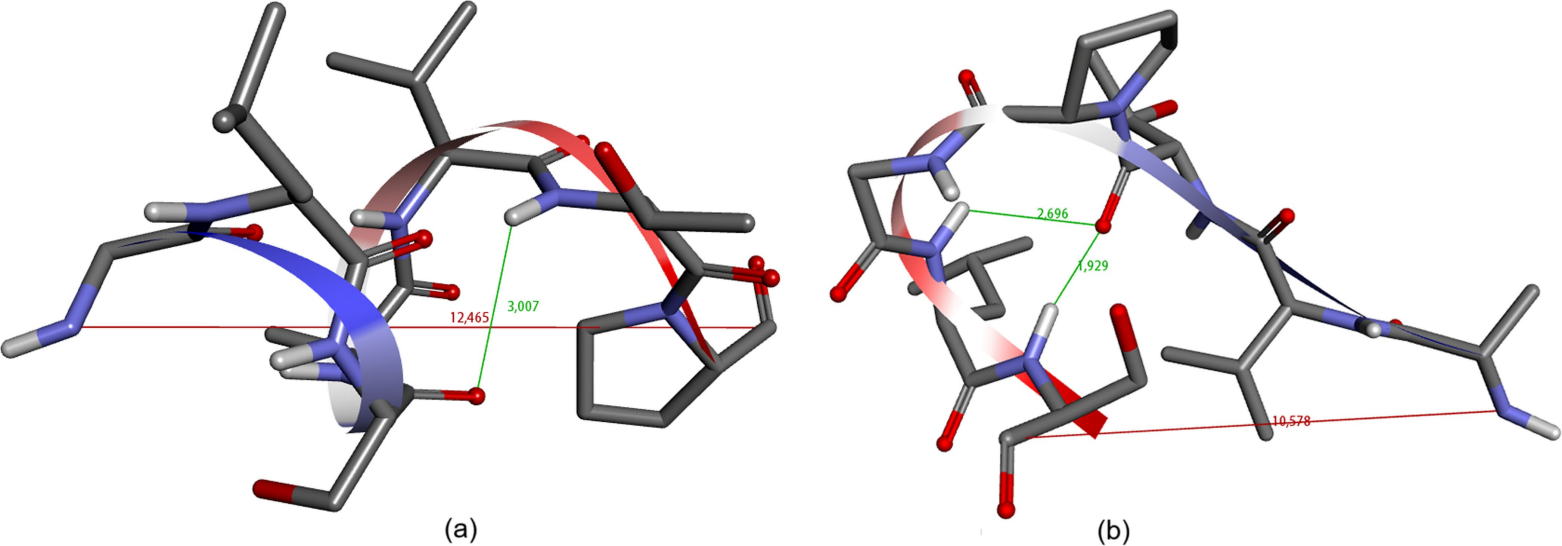
(a) β ‐turn formation of precursor 1 from PEP‐FOLD4; (b) β ‐turn formation of precursor 2 from PEP‐FOLD4.

Cyclization result aligns with the results obtained from PEP‐FOLD 4 prediction. Precursor 1 adopts a beta turn type 1, evidenced by the distance between the carbonyl oxygen and the amide hydrogen being less than 4 Å. However, despite the presence of a β‐turn, the molecule assumes a helical structure due to residue bending in the opposite direction.^[13]^ This results in a considerable distance between the N‐terminal and C‐terminal, posing challenges for cyclization. On the other hand, precursor 2 forms β ‐turn type II and a beta bulge, characterized by two amide hydrogen bonds with the carbonyl oxygen (Figure 2). Although the distance is relatively far, it is not obstructed by spiral geometry and does not bend in the opposite direction like precursor 1. This structural configuration facilitates cyclization, even over an extended period. Both precursor 1 and precursor 2 showed good stability with −5.58315 and −3.43476 sOPEP energy, respectively (S9,S10).^[14]^

The crude cyclic heptapeptide were purified with semipreparative HPLC to obtain peak at 20.3 min (S.12). The target compound **10** was yielded 8.2 %. The mass spectrum of **10** showed *m/z* [M+H^+^] 626.3526 (calculated C_28_H_48_N_7_O_9_ 626.3526) and analytical HPLC revealed the desired peak at 31.7 min (S.13 and S.14). The ^13^C NMR spectrum showed seven carbonyls from 169.03–174.02 ppm, these carbonyls belong to amide carbonyl from seven amino acids. The ^1^H NMR also showed eight α‐proton around 3.38–4.69 ppm. These protons showed all α‐protons, belong to the seven amino acids, including Gly that has two α‐proton. The total assignment of protons and carbons in cyclosenegalin A was achieved using HSQC, HMBC, and TOCSY (S.17–S.20). Weak proton correlations of Thr‐Leu and Pro‐Leu, observed in ROESY spectrum, indicates a conformational fold in cyclosenegalin A (S.21). The comparison of NMR chemical shifts of cyclosenegalin A and the literature is shown in Table 2.

**Table 2:** ^1^H NMR and ^13^C NMR spectra data of cyclosenegalin A compared to isolated cyclosenegalin A by Cen‐Pacheco *et al*., 2019^[1b]^.

Amino acid	Position	Chen Pacheco *et al*., 2019 (C_2_D_6_OS)		Cyclosenegalin A (CD_3_OD)	
		δC (ppm)	δH (ppm, m, *J* in Hz)	δC (ppm)	δH (ppm, m, *J* in Hz)
Pro	CO	174.4		172.7	
	αCH	63.1	4.28 (1H, m)	61.6	4.18 (1H, m)
	βCH_2_	30.5	1.99 (1H, m)	29.0	1.87 (1H, m)
			2.26 (1H, m)		2.16 (1H, m)
	γCH_2_	26	2.00 (1H, m)	24.5	1.90 (1H, m)
			2.16 (1H, m)		2.06 (1H, m)
	δCH_2_	49.8	3.74 (1H, ddd, *J*=7.0, 9.7, 10.0)	48.3	3.63 (1H, m)
			4.02, (1H, ddd, *J*=2.9, 7.8, 10.0)		3.92 (1H, m)
Gly	CO	171.3		170.2	
	αCH_2_	43.9	3.48 (1H, dd, *J*=4.0, 17.0)	42.4	3.38 (1H, d, *J*=17.1)
			4.34 (1H, dd, *J*=8.5, 17.0)		4.24 (1H, d, *J*=17.1)
	NH		8.89, (1H, dd, *J*=4.0, 8.5)		
Leu	CO	174.0		171.9	
	αCH	54.4	4.74, (1H, dd, *J*=5.9, 10.4)	53.0	4.65 (1H, m)
	βCH_2_	44.9	1.53 (2H, m)	43.6	1.43 (2H, m)
	γCH	25.9	1.61 (1H, dt, *J*=6.7, 13.3)	24.5	1.51 (1H, tt, *J*=13.1, 6.2)
	δCH_3_	22.2	0.95 (3H, d, *J*=6.7)	20.7	0.82 (3H, d, *J*=6.2)
	δ′CH_3_	23.3	0.95 (3H, d, *J*=6.7)	21.9	0.84 (3H, d, *J*=6.2)
	NH		8.16 (1H, d, *J*= 10.4)		
Ser	CO	171.6		169.9	
	αCH	55.6	4.70 (1H, ddd, *J*=1.5, 2.9, 7.2)	54.2	4.59 (m, 1H)
	βCH_2_	64.7	3.97 (1H, dd, *J*=1.5, 10.9)	63.2	3.87 (dd, *J*=10.9, 1.6, 1H)
			4.29 (1H, dd, *J*=2.9, 10.9)		4.19 (1H, m)
	NH		8.77 (1H, d, *J*=7.2)		
Ala	CO	175.4		173.0	
	αCH	52.9	4.18 (1H, q, *J*=7.4)	51.4	4.08 (1H, q, *J*=7.4)
	βCH_3_	17.1	1.46 (3H, d, *J*=7.4)	15.7	1.36 (3H, d, *J*=7.4)
	NH		8.76 (1H, m)		
Val	CO	173.3		174.0	
	αCH	60.5	4.34 (1H, d, *J*=6.5)	59.1	4.25 (1H, m)
	βCH	32.1	2.14 (1H, m)	30.6	2.04 (1H, m)
	γCH_3_	18.6	0.92 (3H, d, *J*=6.8)	17.1	0.84 (3H, d, *J*=6.1,)
	γ′CH_3_	19.8	0.94 (3H, d, *J*=6.8)	18.3	0.85 (3H, d, *J*=6.1).
	NH		7.60 (1H, d, *J*=10.2)		
Thr	CO	170.5		169.0	
	αCH	57.9	4.78 (1H, dd, *J*=9.0, 9.4)	56.5	4.69 (1H, t, *J=9*.27)
	βCH	69.2	3.87 (1H, dq, *J*=6.3, 9.0)	67.8	3.75 (1H, dq, *J*=9.27, 6.4)
	γCH_3_	20.6	1.22 (3H, d, *J*=6.3)	19.1	1.13 (3H, d, *J*=6.4)
	NH		7.23 (1H, d, *J*=9.4)		

Cyclosenegalin A was evaluated for its antimicrobial properties against *Staphylococcus aureus* ATCC 6538, *Staphylococcus epidermidis* ATCC 12228, *Escherichia coli* ATCC 11229*, Salmonella thypimurium* ATCC 14028, and *Candida albicans* ATCC 10231 with Ciprofloxacin or Nystatin as positive controls. Cyclosenegalin A showed MIC >1000 ppm and was considered as inactive, while the positive control showed MIC of 0.156, 0.039, 0.005, 0.039 and 15.625 ppm, respectively.

## Conclusions

Total synthesis of cyclosenegalin A was successfully conducted through a combination of solid‐ and liquid‐phase method with 8.2 % yield. β‐turn type II in cyclosenegalin A facilitate the head to tail cyclization process. Cyclosenegalin A showed no activity against *Staphylococcus aureus* ATCC 6538, *Staphylococcus epidermidis* ATCC 12228, *Escherichia coli* ATCC 11229*, Salmonella thypimurium* ATCC 14028, and *Candida albicans* ATCC 1023.

## Supporting Information

The authors have cited additional references within the Supporting Information. Figure S.1–S.6 Spectroscopic characterization of linear precursor, Figure S.7–S.8 Spectroscopic characterization of compound **9**, Figure S.9–S.10. PEP‐FOLD4 coordinate, Figure S.11–S.12 Cyclization HPLC chromatogram, Figure S.13–S.21 Spectroscopic characterization of compound **10**.

## Conflict of Interests

The authors declare no conflict of interest.

1

## Supporting information

As a service to our authors and readers, this journal provides supporting information supplied by the authors. Such materials are peer reviewed and may be re‐organized for online delivery, but are not copy‐edited or typeset. Technical support issues arising from supporting information (other than missing files) should be addressed to the authors.

Supporting Information

## Data Availability

The data that support the findings of this study are available in the supplementary material of this article.
