# Supplementary material for: Total Synthesis of Cyclosenegalin A
Source: ChemistryOpen. 2024 Aug 20;13(12):e202400175. doi: 10.1002/open.202400175 (PMC12056923; doi:10.1002/open.202400175)
Supplement: Supplementary file 1 — Supporting Information [file OPEN-13-e202400175-s001.pdf]

ChemistryOpen

Supporting Information

Total Synthesis of Cyclosenegalin A

Anderson Arnold Aloanis, Tati Herlina, Ari Hardianto, and Rani Maharani*

ChemistryOpen

Supporting Information

Total Synthesis of Cyclosenegalin A

Anderson Arnold Aloanis[1,2], Tati Herlina[1], Ari Hardianto[1], Rani Maharani[1,3*]

[1]Department of Chemistry, Faculty of Mathematics and Natural Sciences, Universitas Padjadjaran, Jalan Raya Bandung Sumedang Km 21 Jatinangor 45363 Kabupaten Sumedang West Java Indonesia

[2]Department of Chemistry, Faculty of Mathematics and Natural Sciences, Universitas Negeri Manado, Jalan Raya Kampus Unima Tondano 95619 Kabupaten Minahasa North Sulawesi Indonesia

[3]Laboratorium Sentral, Universitas Padjadjaran, Jalan Raya Bandung Sumedang Km 21 Jatinangor 45363 Kabupaten Sumedang West Java Indonesia

*Corresponding author: r.maharani@unpad.ac.id

Contents

S 1. HR TOF-MS spectra of crude linear heptapeptide sequence 1 2

S 2. Calculate mass of crude linear heptapeptides sequence 1 2

S 3. HR TOF-MS spectra of crude linear heptapeptide sequence 2 3

S 4. Calculate mass of crude linear heptapeptides sequence 2 3

S 5. Analytical RP-HPLC chromatogram of crude linear heptapeptide sequence 1 4

S 6. Analytical RP-HPLC chromatogram of crude linear heptapeptide sequence 2 4

S 7. ^{13}C NMR of compound 9 5

S 8. ^{1}H NMR of compound 9 5

S 9. Analytical HPLC of crude cyclopeptide 6

S 10. Analytical HPLC of crude cyclopeptide after -tBu removed 6

S 11. PEP-FOLD4 3D coordinate of precursor 1 7

S 12. PEP-FOLD4 3D coordinate of precursor 2 8

S 13. Analytical RP-HPLC chromatogram of cyclosenegalin A 9

S 14. HRMS spectra of cyclosenegalin A 9

S 15. ^{1}H-NMR spectra of cyclosenegalin A (A) 0.0-10.00 ppm, (B) 0.80-2.30 ppm, (C) 3.00-5.00 ppm 10

S 16. ^{13}C-NMR spectra of cyclosenegalin A 11

S 17. HSQC NMR spectra of cyclosenegalin A 11

S 18. HMBC NMR spectra of cyclosenegalin A 12

S 19. TOCSY spectra of Val, Ala, Leu, and Ser 13

S 20. TOCSY spectra of Gly, Pro, and Thr 14

S 21. ROESY NMR spectra of cyclosenegalin A 15

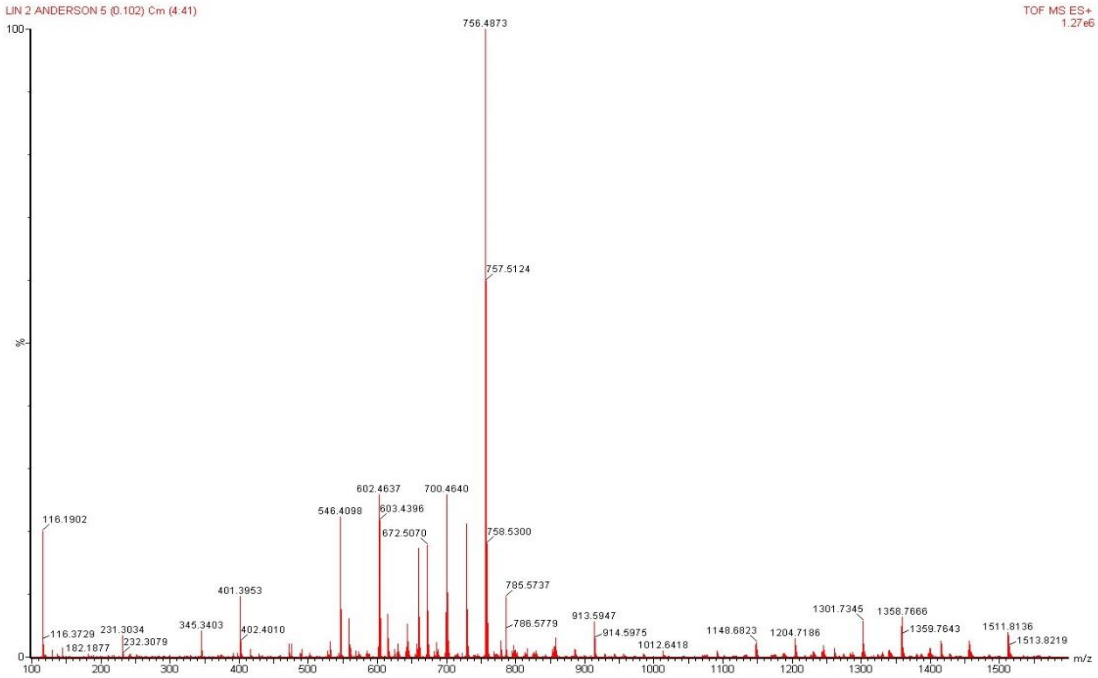

S 1. HR TOF-MS spectra of crude linear heptapeptide sequence 1

Single Mass Analysis

Tolerance = 50.0 mDa / DBE: min = -5.0, max = 50.0
Element prediction: Off
Number of isotope peaks used for i-FIT = 3

Monoisotopic Mass, Even Electron Ions
7590 formula(e) evaluated with 338 results within limits (up to 50 closest results for each mass)
Elements Used:
C: 0-500 H: 0-1000 N: 0-200 O: 0-200
LIN 2 ANDERSON 5 (0.102) Cm (4:41)
TOF MS ES+

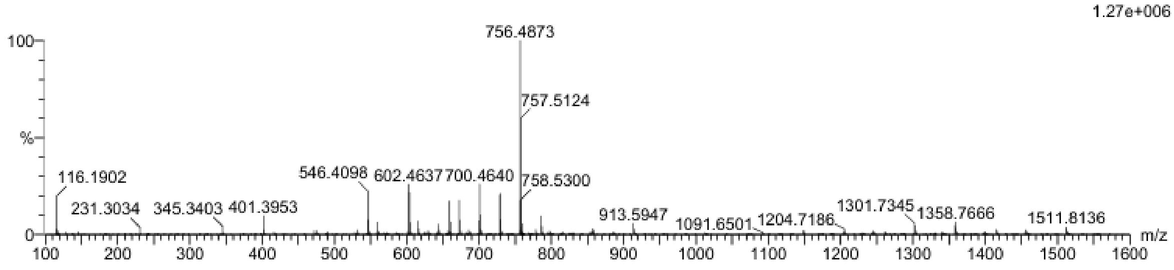

Mass	Calc. Mass	mDa	PPM	DBE	i-FIT	i-FIT (Norm)	Formula
Minimum:				-5.0			
Maximum:		50.0	10.0	50.0			
756.4873	756.4871	0.2	0.3	7.5	211.0	11.4	C36 H66 N7 O10
	756.4871	0.2	0.3	18.5	211.1	11.4	C34 H54 N21
	756.4876	-0.3	-0.4	11.5	213.9	14.2	C19 H50 N33 O
	756.4876	-0.3	-0.4	0.5	213.7	14.1	C21 H62 N19 O11

S 2. Calculate mass of crude linear heptapeptides sequence 1

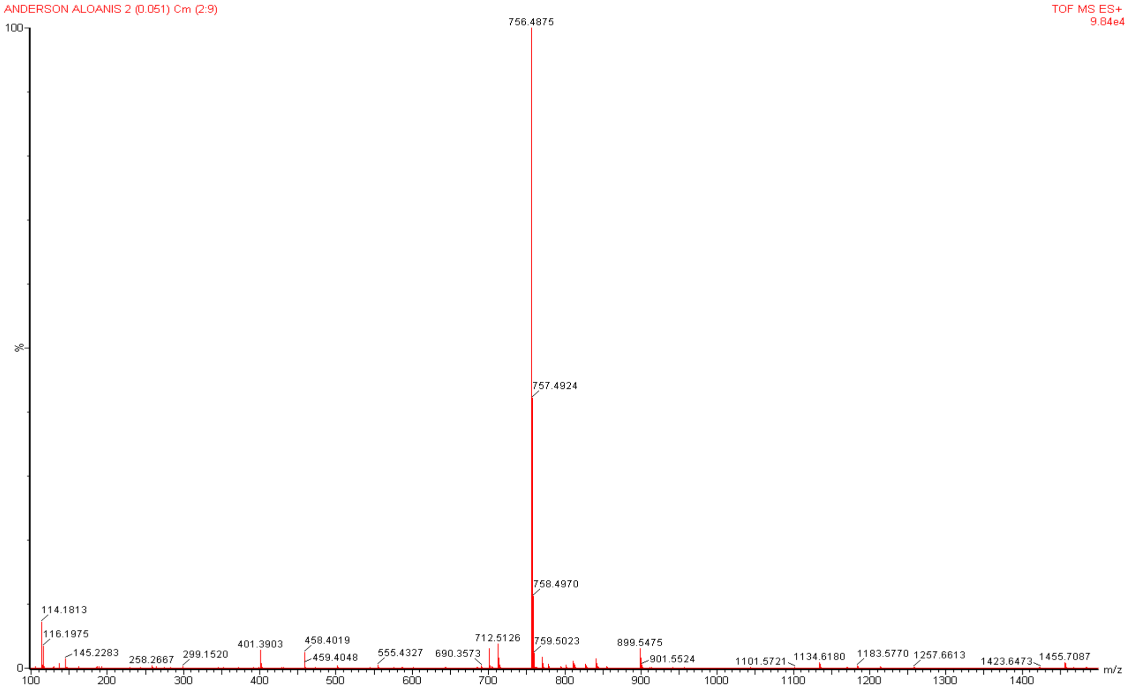

S 3. HR TOF-MS spectra of crude linear heptapeptide sequence 2

Single Mass Analysis

Tolerance = 50.0 mDa / DBE: min = -5.0, max = 50.0
Element prediction: Off
Number of isotope peaks used for i-FIT = 3

Monoisotopic Mass, Even Electron Ions
7590 formula(e) evaluated with 338 results within limits (up to 50 closest results for each mass)
Elements Used:
C: 0-500 H: 0-1000 N: 0-200 O: 0-200
ANDERSON ALOANIS 2 (0.051) Cm (2:9)
TOF MS ES+

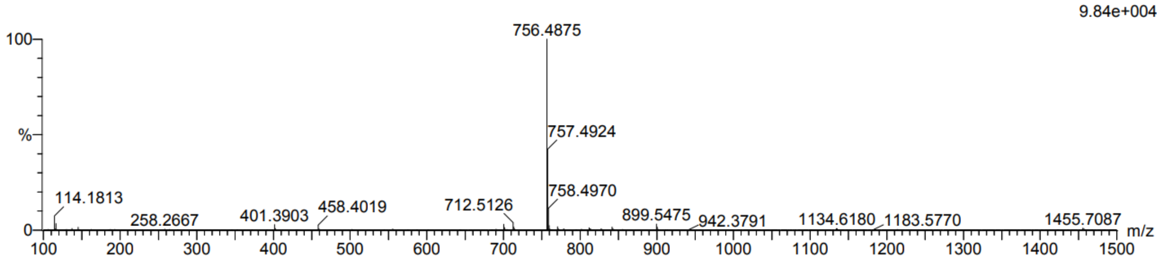

Minimum:				-5.0			
Maximum:		50.0	10.0	50.0			
Mass	Calc. Mass	mDa	PPM	DBE	i-FIT	i-FIT (Norm)	Formula
756.4875	756.4876	-0.1	-0.1	0.5	120.3	13.9	C21 H62 N19 O11
	756.4876	-0.1	-0.1	11.5	121.6	15.2	C19 H50 N33 O
	756.4871	0.4	0.5	7.5	110.6	4.2	C36 H66 N7 O10
	756.4871	0.4	0.5	18.5	115.5	9.1	C34 H54 N21
	756.4881	-0.6	-0.8	4.5	126.4	20.0	C4 H46 N45 O2
	756.4868	0.7	0.9	-0.5	126.1	19.7	C3 H50 N41 O6

S 4. Calculate mass of crude linear heptapeptides sequence 2

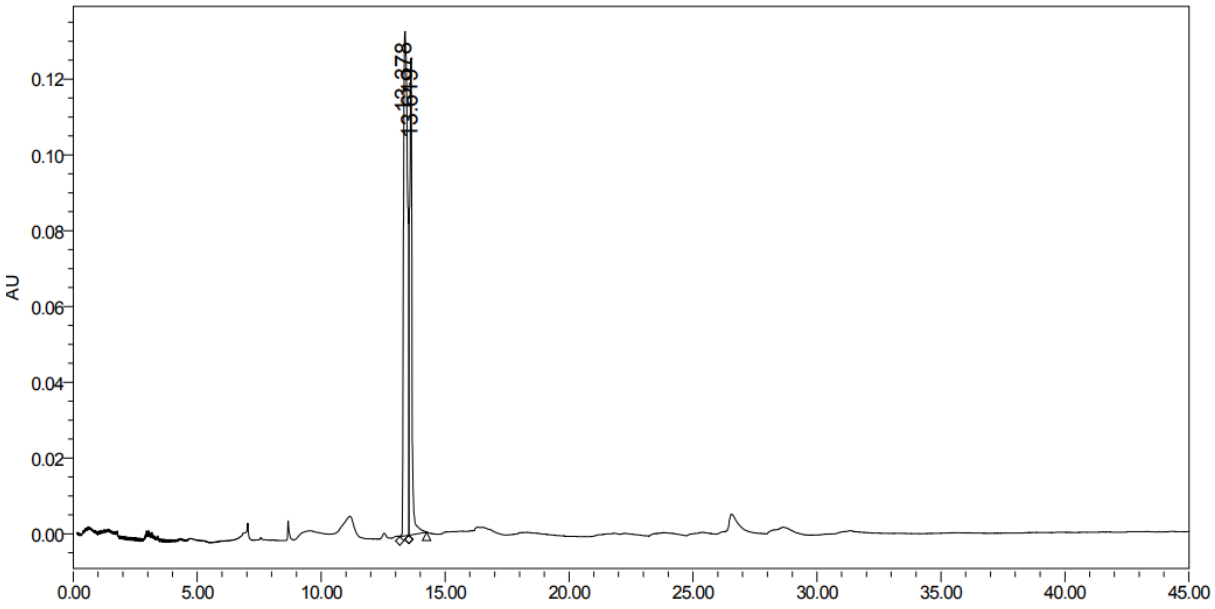

S 5. Analytical RP-HPLC chromatogram of crude linear heptapeptide sequence 1

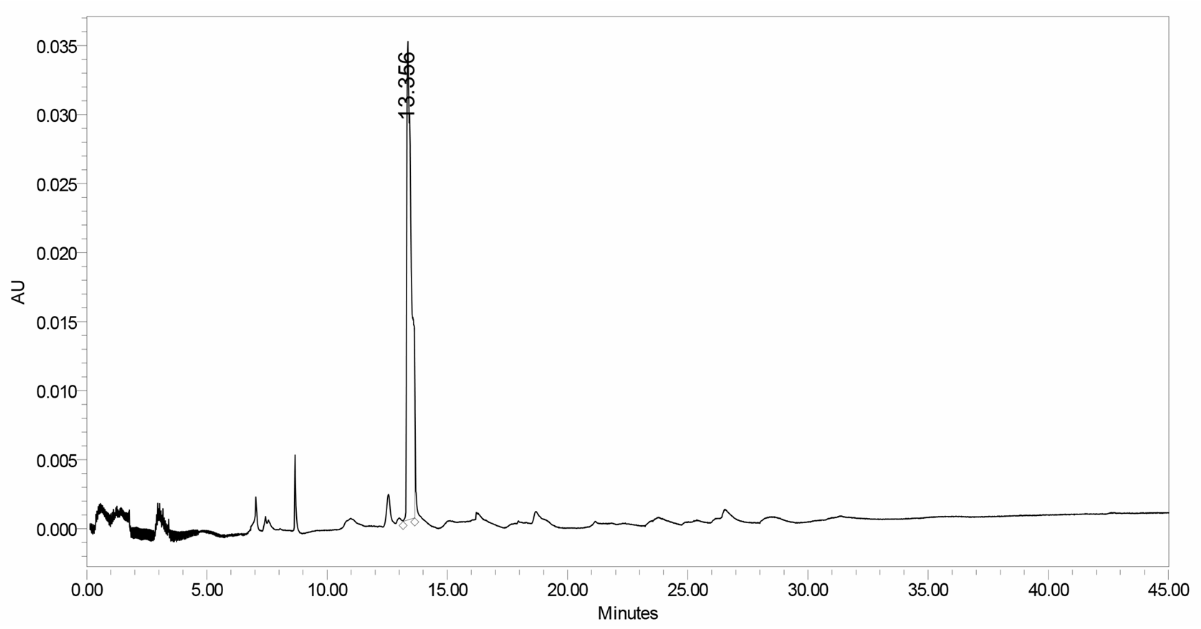

S 6. Analytical RP-HPLC chromatogram of crude linear heptapeptide sequence 2

Compound 9. HRMS: *m/z* [M+H$^+$] 756.4875 (calculated $C_{36}H_{66}N_7O_{10}$ 756.4871). ^{1}H NMR (700 MHz, MeOD) δ = 4.66 (d, *J*=6.1, 1H), 4.57 – 4.51 (m, 1H), 4.46 (t, *J*=4.2, 1H), 4.43 (t, *J*=4.9, 1H), 4.34 (dd, *J*=8.1, 5.8, 1H), 4.23 (dd, *J*=7.3, 0.7, 1H), 4.00 – 3.92 (m, 3H), 3.87 – 3.82 (m, 2H), 3.75 (dd, *J*=9.3, 4.4, 2H), 3.68 (d, *J*=16.9, 1H), 3.59 (dd, *J*=9.2, 3.9, 1H), 2.16 (ddt, *J*=12.6, 8.1, 6.8, 1H), 2.10 – 2.04 (m, 1H), 2.04 – 1.99 (m, 1H), 1.99 – 1.94 (m, 1H), 1.94 – 1.88 (m, 1H), 1.64 – 1.62 (m, 1H), 1.57 – 1.50 (m, 2H), 1.45 (d, *J*=7.0, 3H), 1.16 (s, 9H), 1.13 (s, 9H), 1.11 (d, *J*=6.2, 3H), 0.92 – 0.86 (m, 12H). ^{13}C NMR (176 MHz, MeOD) δ 173.2, 172.9, 171.8, 171.4, 169.6, 169.6, 169.3, 74.7, 73.2, 68.2, 61.3, 60.6, 58.9, 55.7, 53.2, 51.5, 48.7, 48.1, 41.9, 40.7, 30.3, 29.7, 27.4, 26.3, 24.5, 24.5, 22.0, 21.0, 18.4, 18.3, 17.3, 16.5.

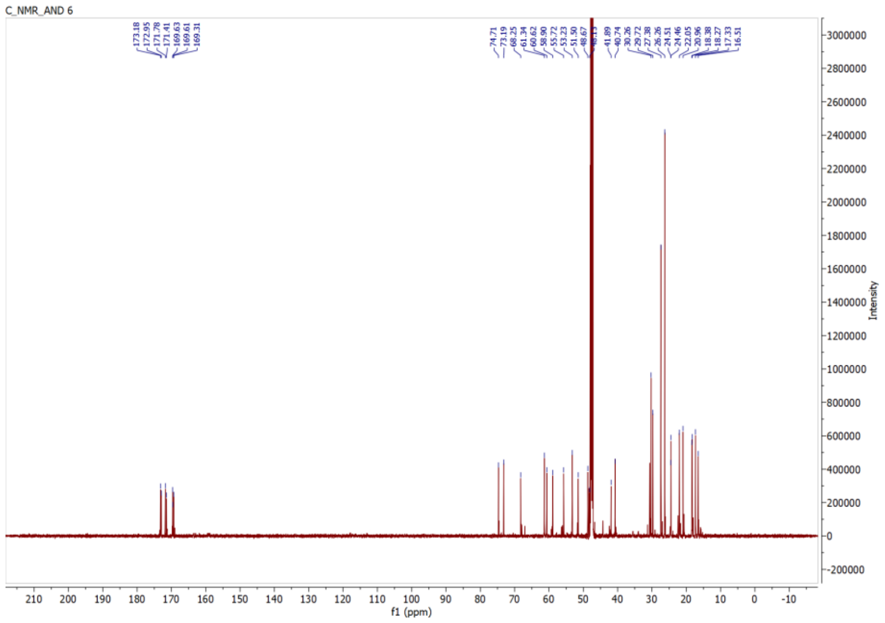

S 7. ^{13}C NMR of compound **9**

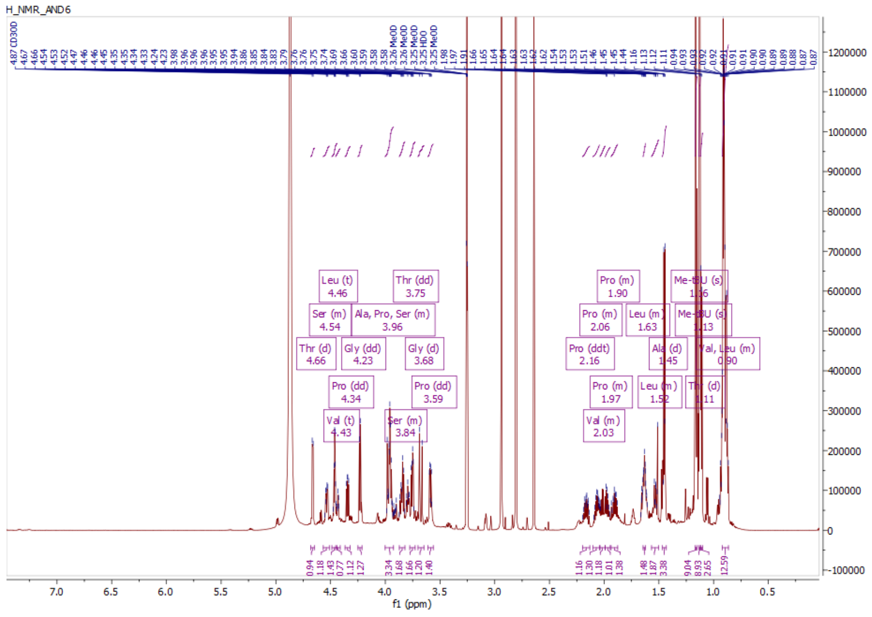

S 8. ^{1}H NMR of compound **9**

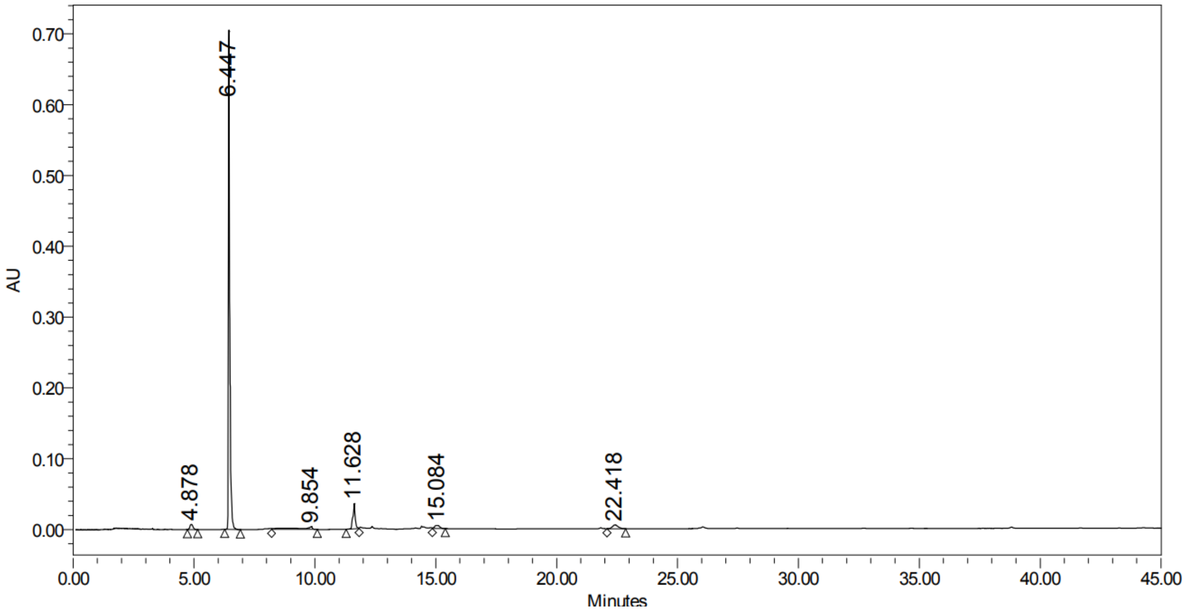

S 9. Analytical HPLC of crude cyclopeptide

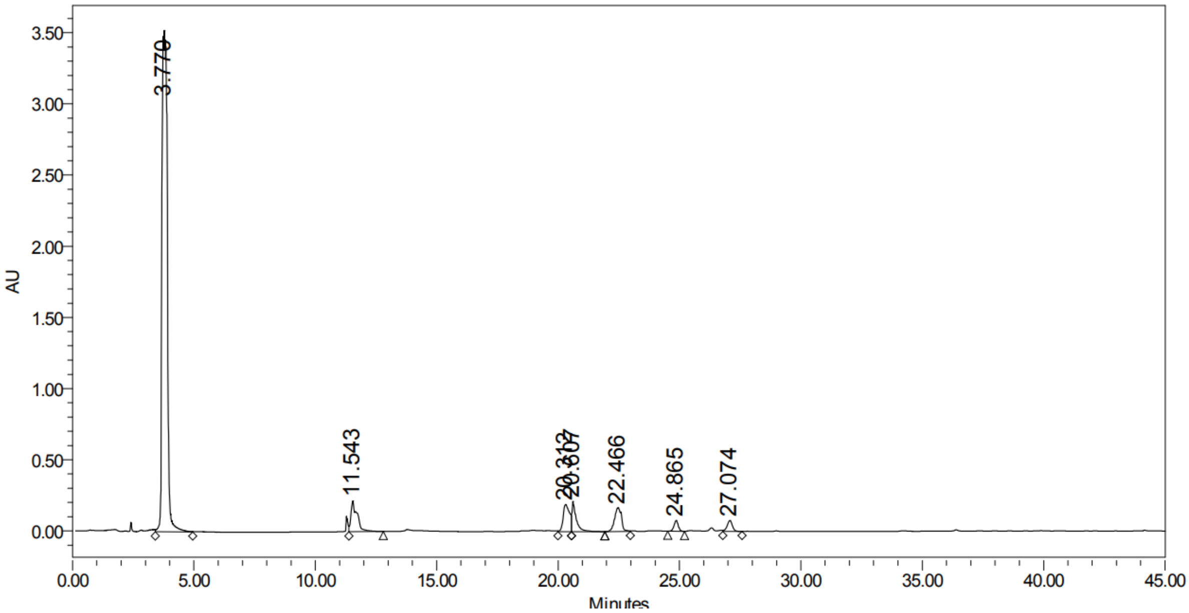

S 10. Analytical HPLC of crude cyclopeptide after -tBu removed

```
HEADER   PEPFOLD-00055_bestene1-mc.pdb
REMARK   Component boundaries: 0: 1 7

REMARK   SA Trajectory: aVZZ
REMARK      eCACA       eHb   ePhiPos  eVdWBBBB  eVdWBBSC  eVdWSCSC     Total
REMARK     -0.037    -3.619     0.157    -0.352    -0.134    -1.598    -5.583
REMARK    sOPEP Energy: -5.58315
MODEL         1
ATOM      0  N   GLY A   1      58.870 -89.605 -50.799
ATOM      1  CA  GLY A   1      58.875 -88.294 -51.411
ATOM      2  C   GLY A   1      59.003 -87.223 -50.358
ATOM      3  O   GLY A   1      59.955 -86.452 -50.371
ATOM      4  H   GLY A   1      58.790 -90.439 -51.380
ATOM      0  N   LEU A   2      58.050 -87.164 -49.440
ATOM      1  CA  LEU A   2      58.129 -86.154 -48.401
ATOM      2  C   LEU A   2      59.531 -86.136 -47.836
ATOM      3  O   LEU A   2      60.159 -85.082 -47.731
ATOM      4  H   LEU A   2      57.303 -87.793 -49.460
ATOM      5  CB  LEU A   2      57.126 -86.486 -47.280
ATOM      6  CG  LEU A   2      55.690 -86.473 -47.777
ATOM      7  CD1 LEU A   2      54.725 -87.033 -46.746
ATOM      8  CD2 LEU A   2      55.218 -85.068 -48.117
ATOM      0  N   SER A   3      60.024 -87.318 -47.494
ATOM      1  CA  SER A   3      61.354 -87.472 -46.928
ATOM      2  C   SER A   3      62.387 -86.729 -47.769
ATOM      3  O   SER A   3      63.166 -85.924 -47.251
ATOM      4  H   SER A   3      59.475 -88.115 -47.629
ATOM      5  CB  SER A   3      61.719 -88.968 -46.887
ATOM      6  OG  SER A   3      61.814 -89.436 -48.235
ATOM      0  N   ALA A   4      62.370 -86.998 -49.072
ATOM      1  CA  ALA A   4      63.305 -86.393 -50.018
ATOM      2  C   ALA A   4      63.199 -84.864 -50.029
ATOM      3  O   ALA A   4      64.222 -84.176 -50.007
ATOM      4  H   ALA A   4      61.704 -87.626 -49.412
ATOM      5  CB  ALA A   4      62.997 -86.922 -51.431
ATOM      0  N   VAL A   5      61.980 -84.328 -50.058
ATOM      1  CA  VAL A   5      61.820 -82.877 -50.092
ATOM      2  C   VAL A   5      62.185 -82.148 -48.794
ATOM      3  O   VAL A   5      62.816 -81.092 -48.851
ATOM      4  H   VAL A   5      61.191 -84.905 -50.057
ATOM      5  CB  VAL A   5      60.346 -82.567 -50.413
ATOM      6  CG1 VAL A   5      60.054 -81.076 -50.363
ATOM      7  CG2 VAL A   5      59.949 -83.054 -51.797
ATOM      0  N   THR A   6      61.802 -82.699 -47.647
ATOM      1  CA  THR A   6      62.050 -82.035 -46.375
ATOM      2  C   THR A   6      63.442 -82.002 -45.703
ATOM      3  O   THR A   6      63.558 -81.532 -44.560
ATOM      4  H   THR A   6      61.348 -83.564 -47.657
ATOM      5  CB  THR A   6      61.104 -82.697 -45.356
ATOM      6  OG1 THR A   6      59.754 -82.623 -45.823
ATOM      7  CG2 THR A   6      61.168 -82.021 -43.997
ATOM      0  N   PRO A   7      64.506 -82.443 -46.370
ATOM      1  CA  PRO A   7      65.797 -82.432 -45.705
ATOM      2  C   PRO A   7      66.437 -81.061 -45.787
ATOM      3  O   PRO A   7      66.021 -80.223 -46.570
ATOM      4  CB  PRO A   7      66.667 -83.479 -46.424
ATOM      5  CG  PRO A   7      65.644 -84.402 -47.112
ATOM      6  CD  PRO A   7      64.477 -83.463 -47.425
TER
ENDMDL
```

S 11. PEP-FOLD4 3D coordinate of precursor 1

```
HEADER    PEPFOLD-00002_bestene1-mc.pdb
REMARK    Component boundaries: 0: 1 7

REMARK    SA Trajectory: KKZZ
REMARK       eCACA       eHb   ePhiPos eVdWBBBB eVdWBBSC eVdWSCSC     Total
REMARK      -0.033    -1.282     0.008   -0.201   -0.178   -1.748    -3.435
REMARK     sOPEP Energy: -3.43476
MODEL        1
ATOM      0  N   ALA A   1      -8.014   1.218 -13.049
ATOM      1  CA  ALA A   1      -8.488  -0.135 -12.790
ATOM      2  C   ALA A   1      -9.211  -0.120 -11.450
ATOM      3  O   ALA A   1      -8.713   0.446 -10.473
ATOM      4  H   ALA A   1      -7.499   1.423 -13.906
ATOM      5  CB  ALA A   1      -7.296  -1.108 -12.732
ATOM      0  N   VAL A   2     -10.384  -0.738 -11.404
ATOM      1  CA  VAL A   2     -11.160  -0.757 -10.175
ATOM      2  C   VAL A   2     -11.577  -2.153  -9.752
ATOM      3  O   VAL A   2     -11.585  -3.091 -10.559
ATOM      4  H   VAL A   2     -10.729  -1.185 -12.202
ATOM      5  CB  VAL A   2     -12.430   0.089 -10.383
ATOM      6  CG1 VAL A   2     -13.271   0.175  -9.120
ATOM      7  CG2 VAL A   2     -12.104   1.514 -10.802
ATOM      0  N   THR A   3     -11.925  -2.282  -8.477
ATOM      1  CA  THR A   3     -12.352  -3.558  -7.939
ATOM      2  C   THR A   3     -13.729  -3.874  -8.496
ATOM      3  O   THR A   3     -14.497  -2.970  -8.837
ATOM      4  H   THR A   3     -11.893  -1.501  -7.889
ATOM      5  CB  THR A   3     -12.419  -3.481  -6.402
ATOM      6  OG1 THR A   3     -11.123  -3.197  -5.870
ATOM      7  CG2 THR A   3     -12.903  -4.784  -5.787
ATOM      0  N   PRO A   4     -14.027  -5.162  -8.608
ATOM      1  CA  PRO A   4     -15.301  -5.611  -9.141
ATOM      2  C   PRO A   4     -16.355  -5.418  -7.929
ATOM      3  O   PRO A   4     -16.055  -5.544  -6.737
ATOM      4  CB  PRO A   4     -15.151  -7.091  -9.541
ATOM      5  CG  PRO A   4     -13.639  -7.259  -9.781
ATOM      6  CD  PRO A   4     -13.014  -6.261  -8.802
ATOM      0  N   GLY A   5     -17.533  -4.992  -8.336
ATOM      1  CA  GLY A   5     -18.601  -4.669  -7.379
ATOM      2  C   GLY A   5     -18.368  -3.274  -7.569
ATOM      3  O   GLY A   5     -19.402  -2.749  -7.977
ATOM      4  H   GLY A   5     -17.702  -4.890  -9.293
ATOM      0  N   LEU A   6     -17.258  -2.578  -7.313
ATOM      1  CA  LEU A   6     -17.224  -1.130  -7.511
ATOM      2  C   LEU A   6     -17.410  -0.705  -8.950
ATOM      3  O   LEU A   6     -18.191   0.210  -9.256
ATOM      4  H   LEU A   6     -16.460  -3.040  -6.990
ATOM      5  CB  LEU A   6     -15.861  -0.604  -7.023
ATOM      6  CG  LEU A   6     -15.631  -0.899  -5.550
ATOM      7  CD1 LEU A   6     -14.201  -0.603  -5.127
ATOM      8  CD2 LEU A   6     -16.542  -0.075  -4.655
ATOM      0  N   SER A   7     -16.674  -1.363  -9.835
ATOM      1  CA  SER A   7     -16.794  -1.100 -11.262
ATOM      2  C   SER A   7     -18.156  -1.510 -11.789
ATOM      3  O   SER A   7     -18.692  -0.845 -12.664
ATOM      4  H   SER A   7     -16.040  -2.038  -9.524
ATOM      5  CB  SER A   7     -15.708  -1.894 -12.012
ATOM      6  OG  SER A   7     -15.991  -3.287 -11.854
TER
ENDMDL
```

S 12. PEP-FOLD4 3D coordinate of precursor **2**

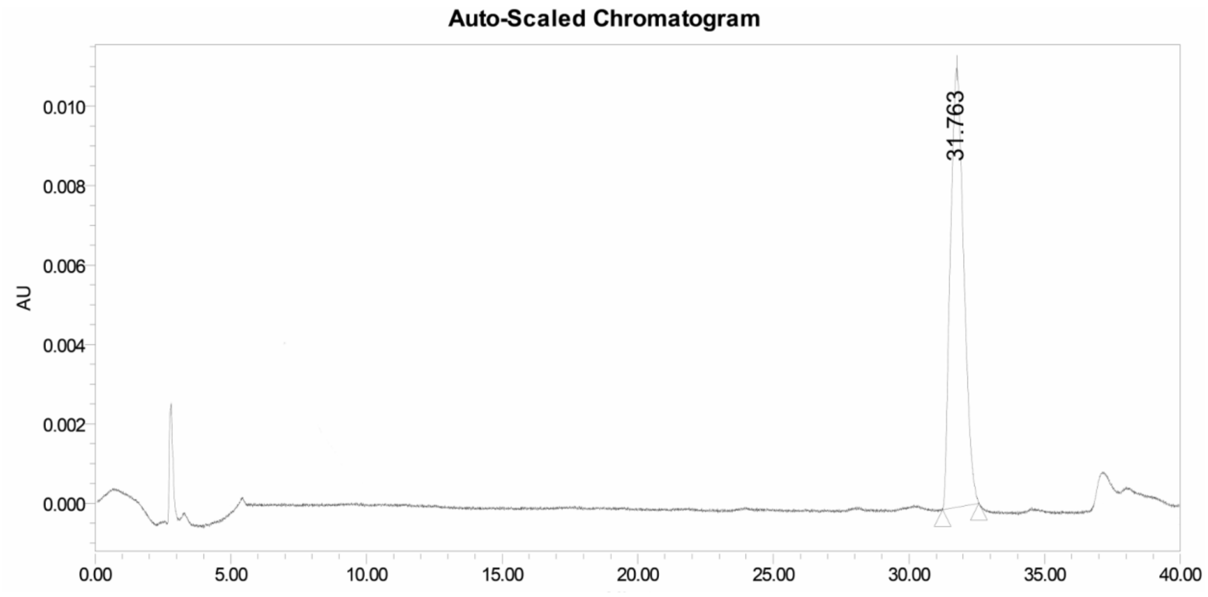

S 13. Analytical RP-HPLC chromatogram of cyclosenegalin A

Elemental Composition Report **Page 1**

Single Mass Analysis
Tolerance = 10.0 PPM / DBE: min = -1.5, max = 50.0
Element prediction: Off
Number of isotope peaks used for i-FIT = 3

Monoisotopic Mass, Even Electron Ions
295 formula(e) evaluated with 1 results within limits (up to 3 best isotopic matches for each mass)
Elements Used:
C: 0-28 H: 0-50 N: 0-7 O: 0-10 I: 0-1

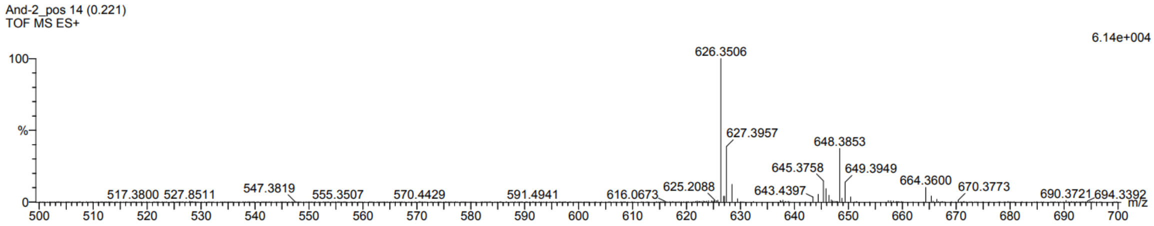

Minimum:				-1.5			
Maximum:		5.0	10.0	50.0			
Mass	Calc. Mass	mDa	PPM	DBE	i-FIT	i-FIT (Norm)	Formula
626.3506	626.3514	-0.8	-1.3	8.5	67.5	0.0	C28 H48 N7 O9

S 14. HRMS spectra of cyclosenegalin A

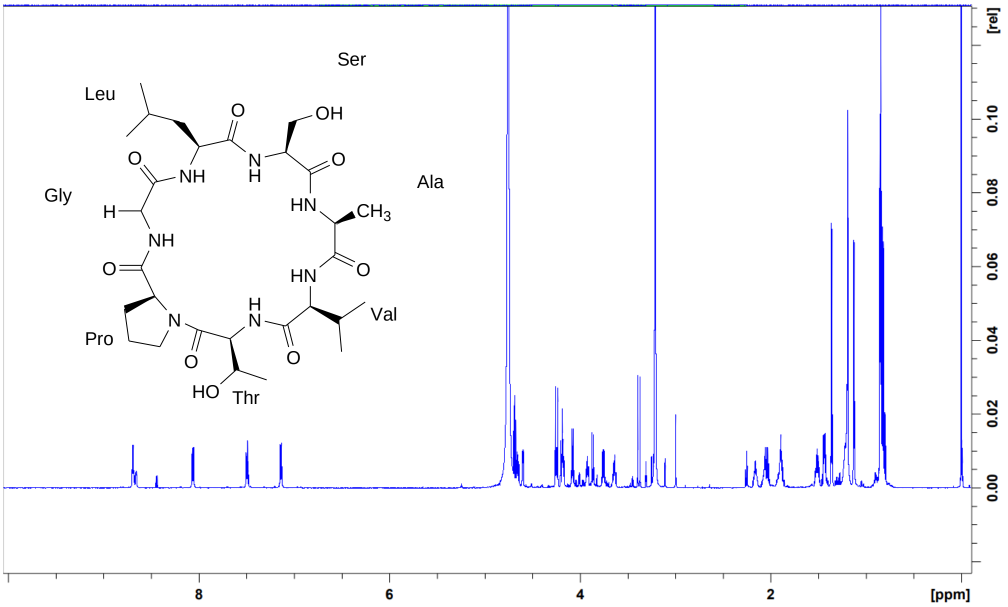

(A)

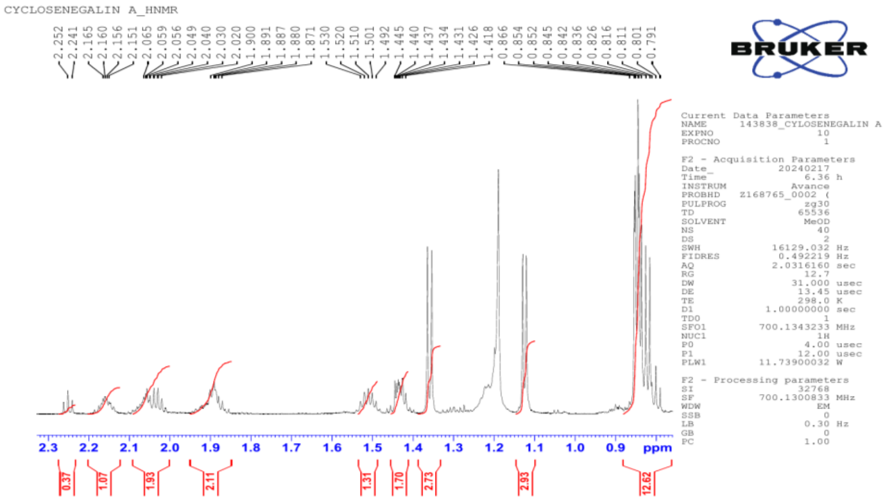

(B)

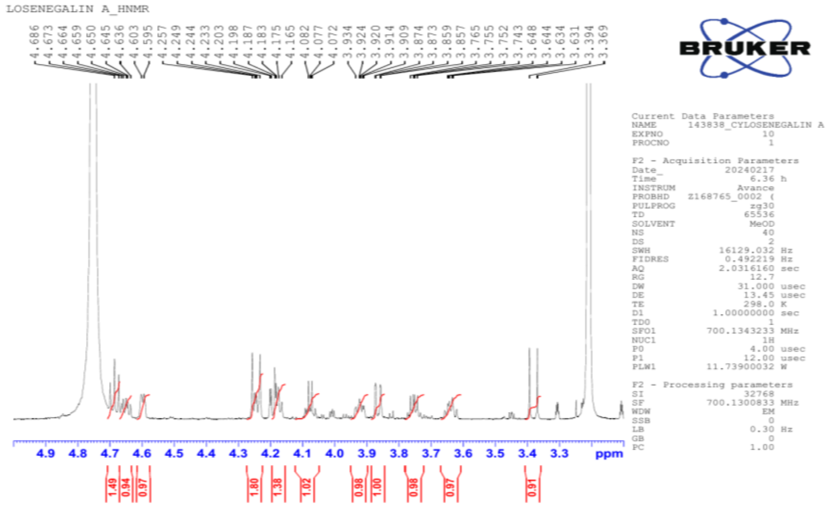

(C)

S 15. ^{1}H-NMR spectra of cyclosenegalin A (A) 0.0-10.00 ppm, (B) 0.80-2.30 ppm, (C) 3.00-5.00 ppm

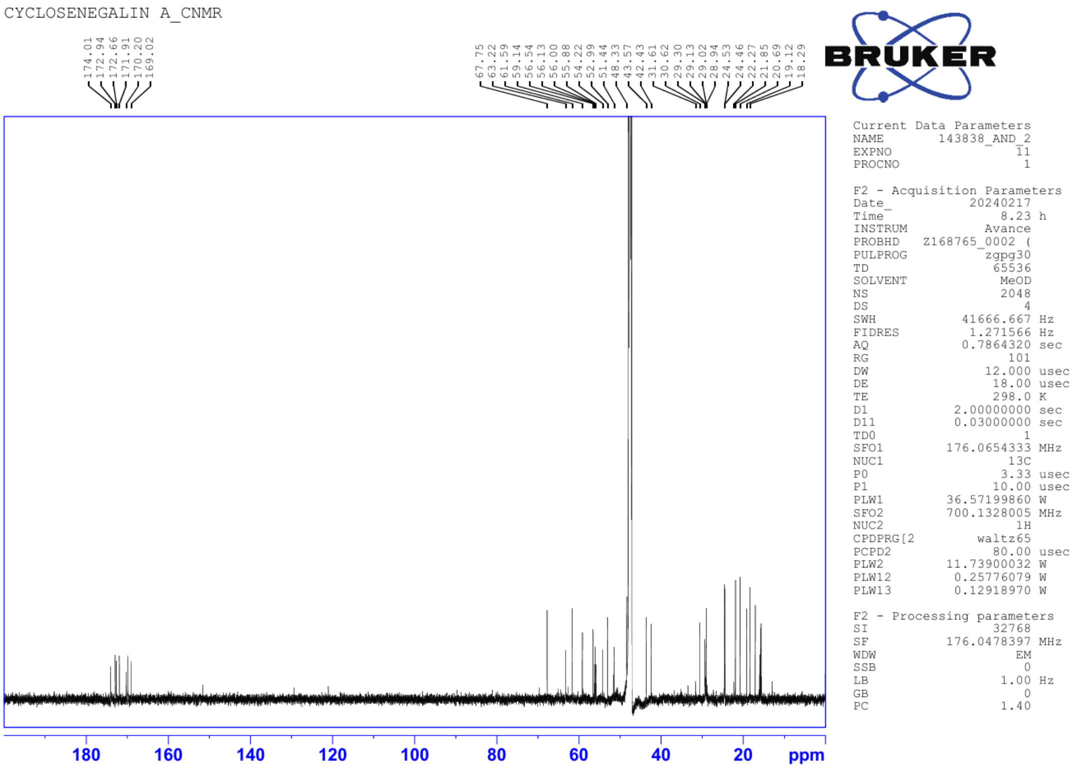

S 16. ^{13}C-NMR spectra of cyclosenegalin A

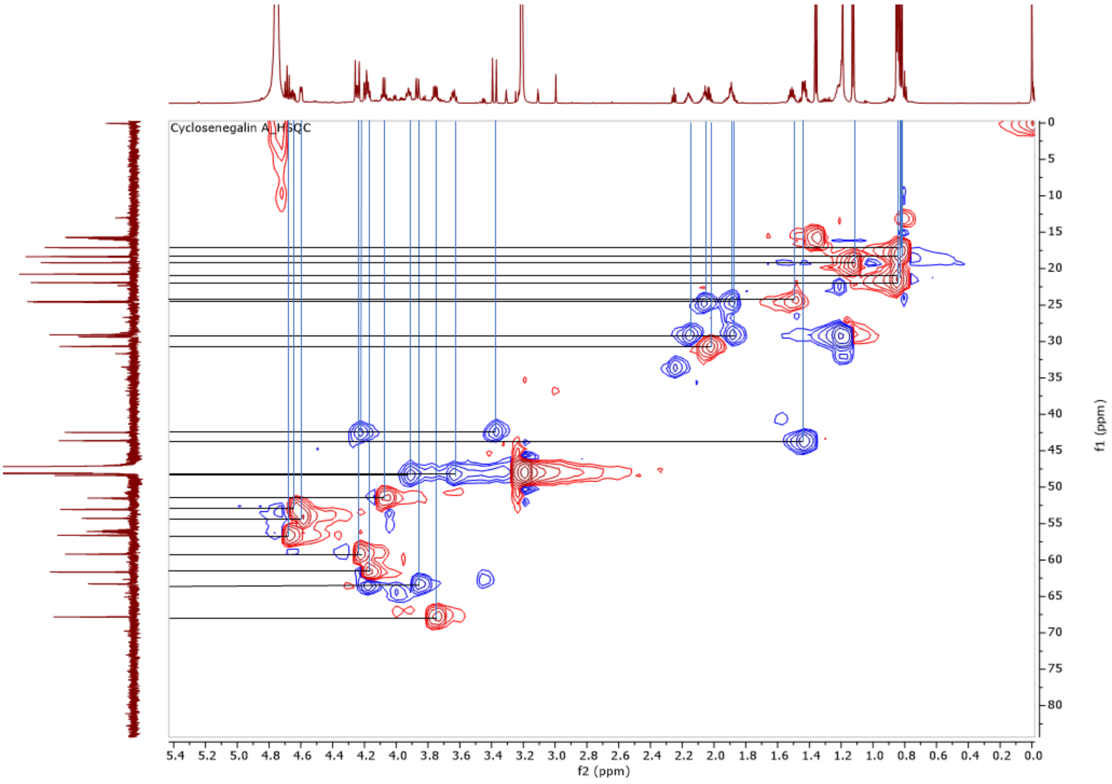

S 17. HSQC NMR spectra of cyclosenegalin A

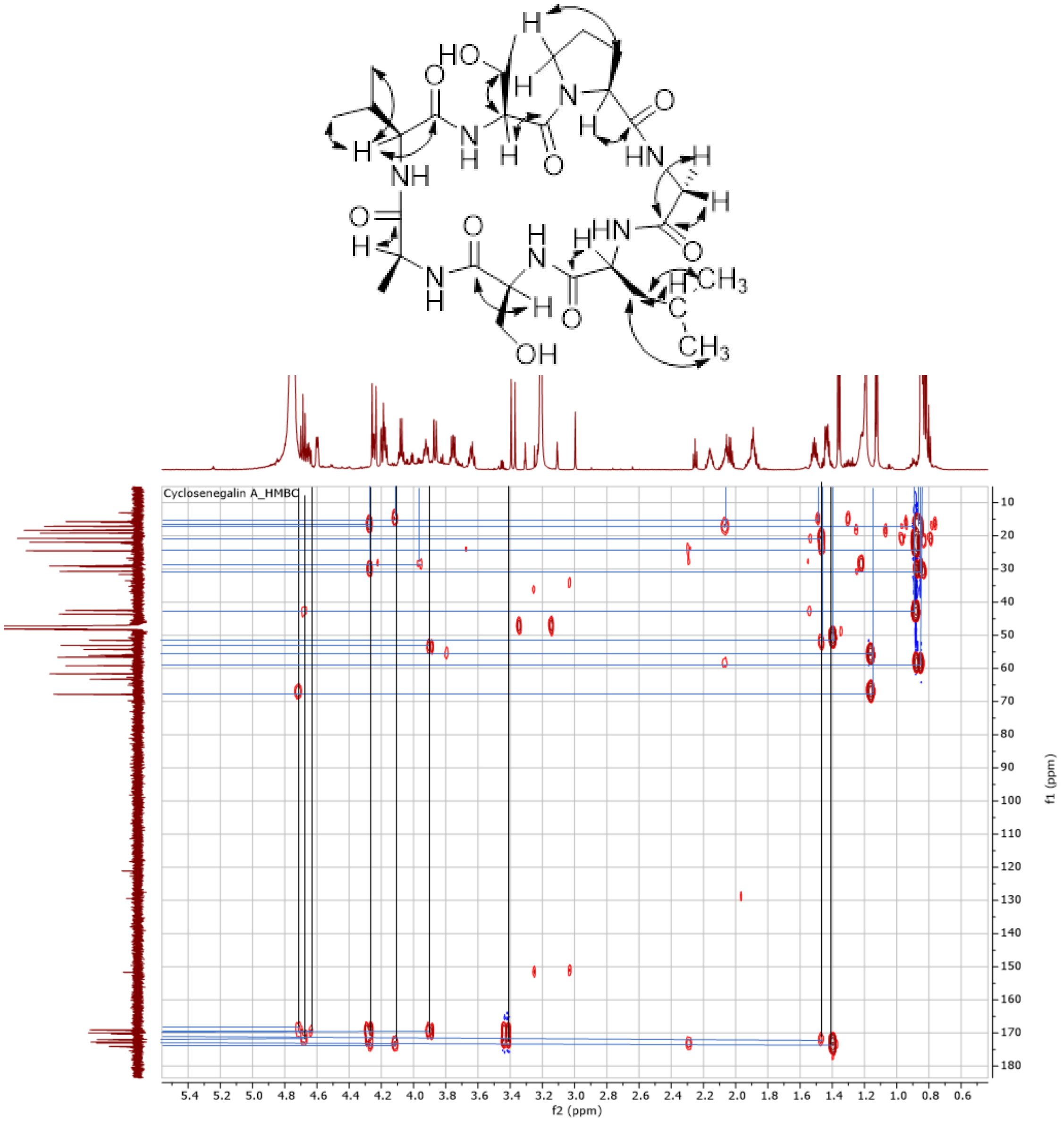

S 18. HMBC NMR spectra of cyclosenegalin A

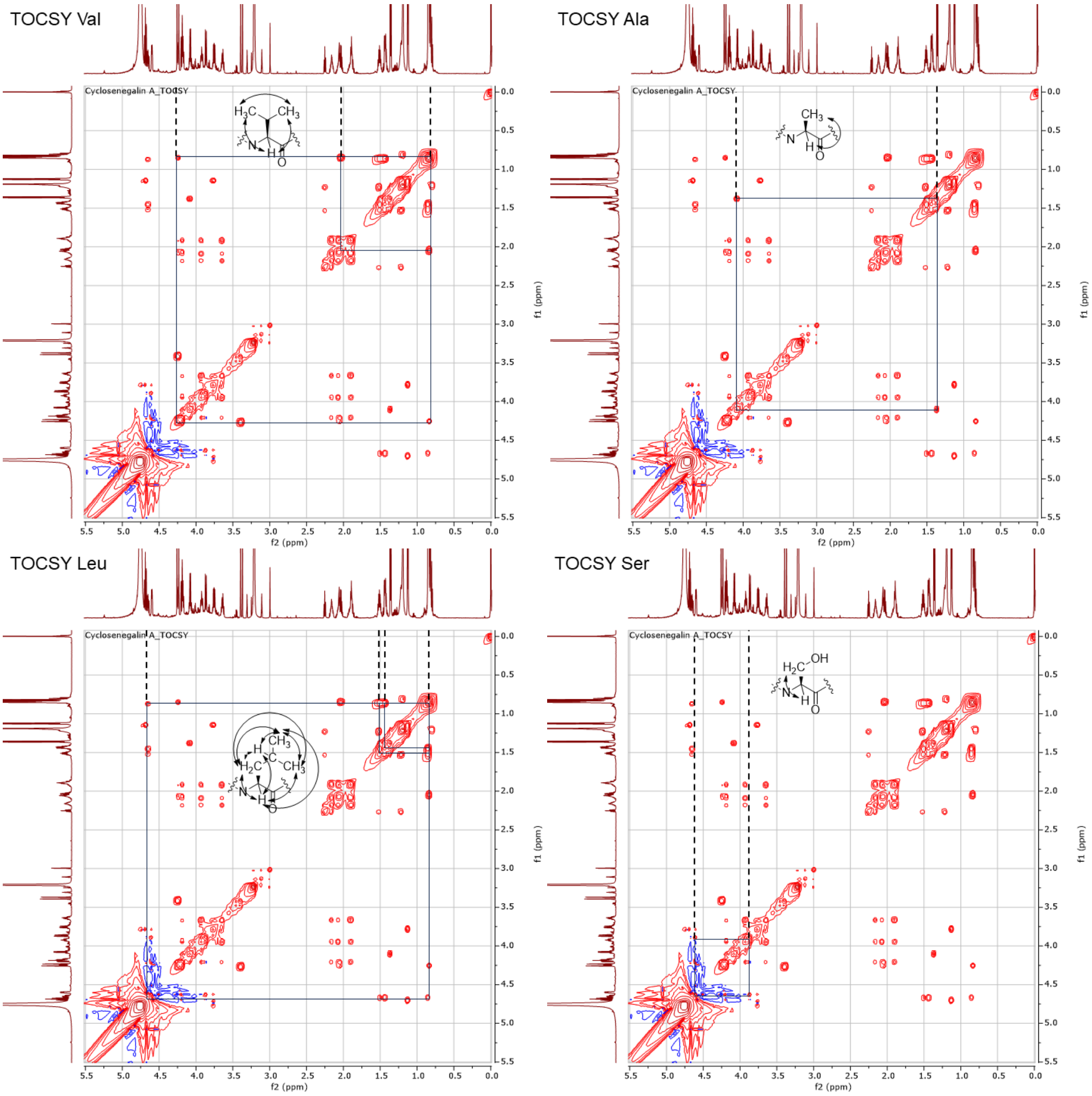

S 19. TOCSY spectra of Val, Ala, Leu, and Ser

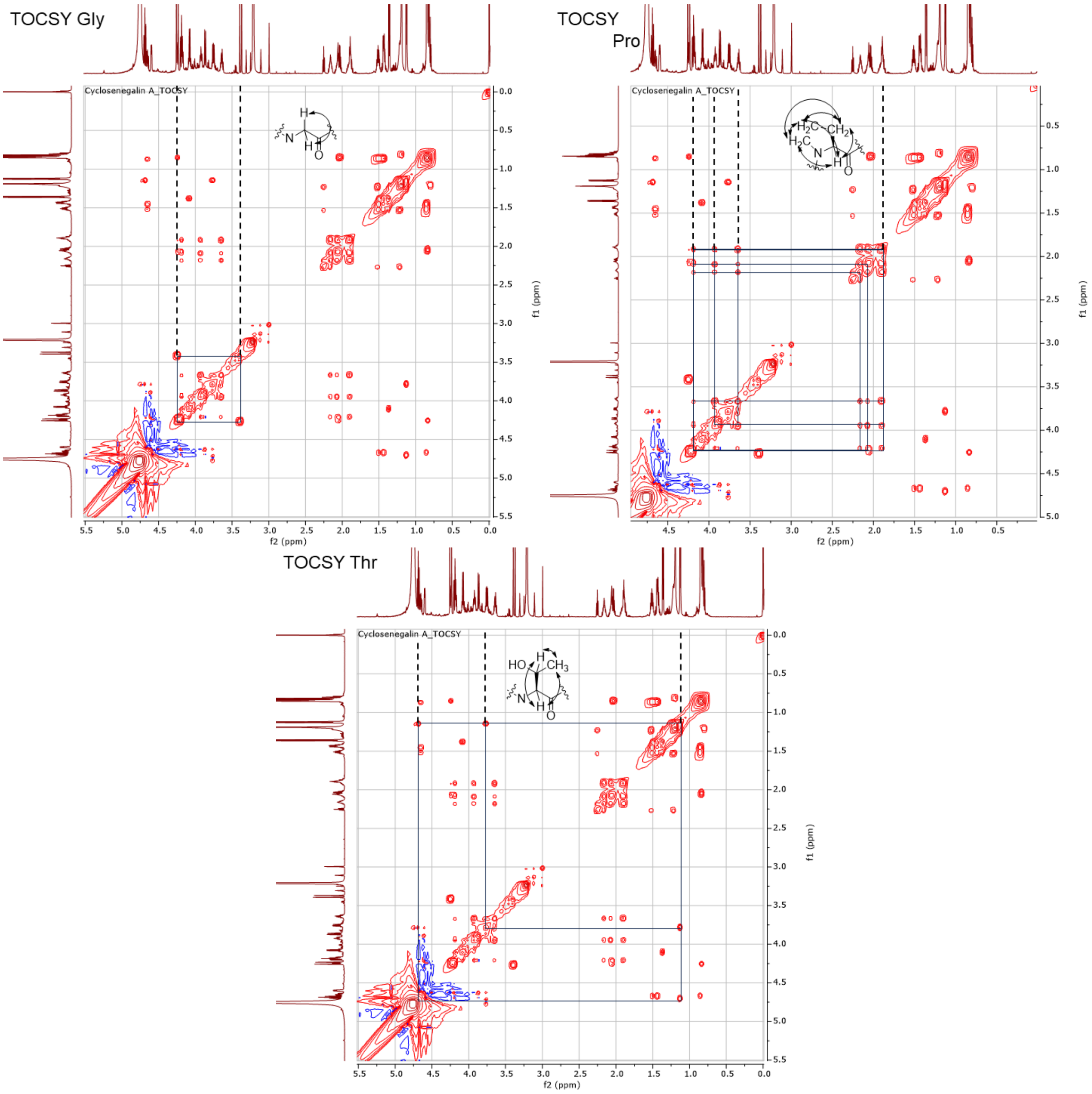

S 20. TOCSY spectra of Gly, Pro, and Thr

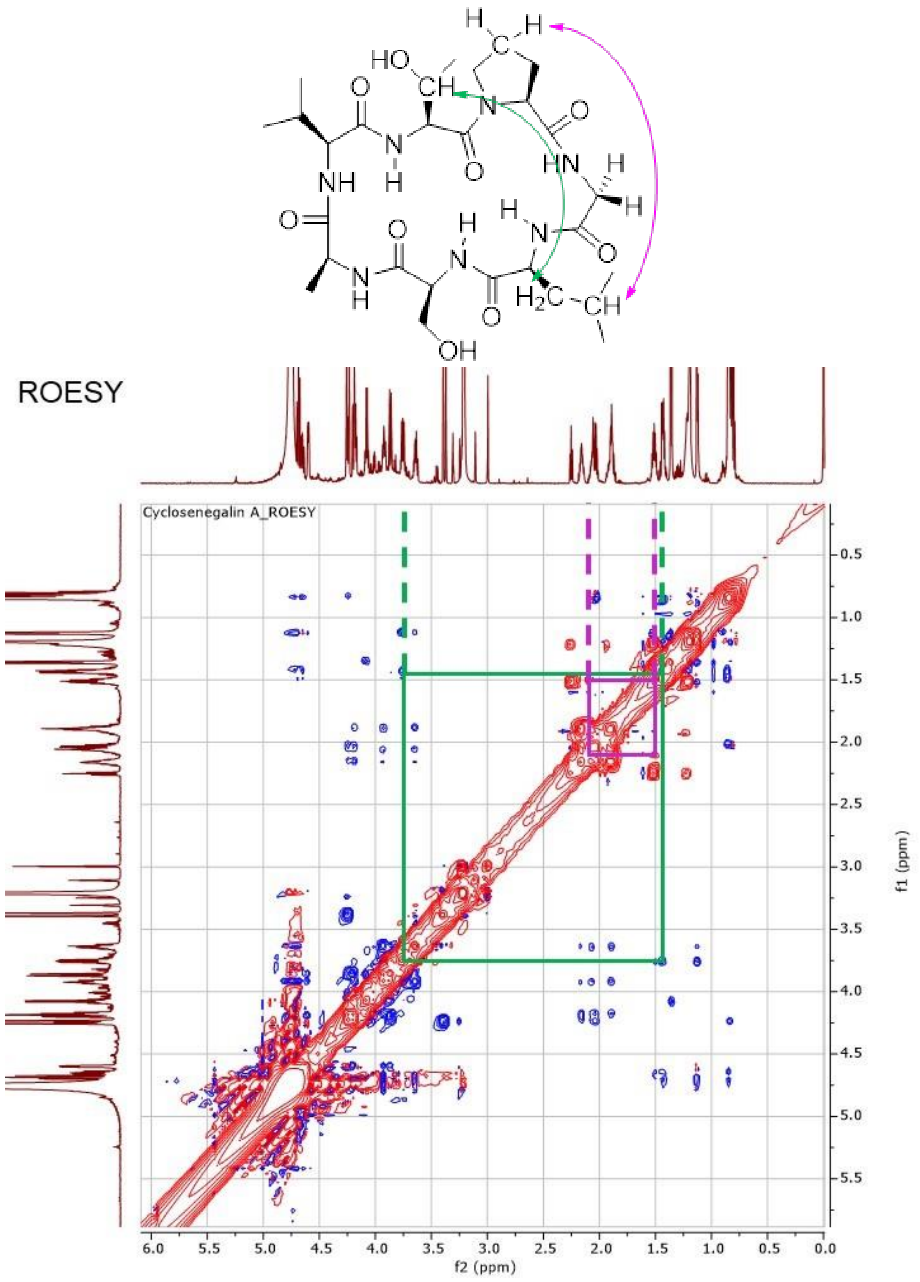

S 21. ROESY NMR spectra of cyclosenegalin A
